# Alopecia in anticoagulated patients

**DOI:** 10.1590/1677-5449.190018

**Published:** 2020-06-08

**Authors:** Marcos Arêas Marques, Carmen Lucia Lascasas Porto, Ana Letícia de Matos Milhomens, Juliana de Miranda Vieira, Claudia Carvalho Alcântara Gomes, Ana Thereza Cavalcanti Rocha, Cíntia da Silva Miller

**Affiliations:** 1 Universidade do Estado do Rio de Janeiro – UERJ, Hospital Universitário Pedro Ernesto, Unidade Docente Assistencial de Angiologia, Rio de Janeiro, RJ, Brasil.; 2 Hospital Barra D’Or, Dermatologia, Rio de Janeiro, RJ, Brasil.; 3 Faculdade de Medicina da Bahia, Departamento de Saúde da Família, Salvador, BA, Brasil.

**Keywords:** alopecia, drug-related side effects and adverse reactions, warfarin, heparin, anticoagulants, rivaroxaban

## Abstract

Alopecia is a common complication of anticoagulant therapy that may have important psychological repercussions for patients, especially female patients, and can interfere with the decision to extend anticoagulation. This review aims to describe the mechanisms potentially involved in the genesis of alopecia during anticoagulant therapy, since these are not yet fully understood, and discusses the existing therapies for the most appropriate management.

## INTRODUCTION

Hair loss is a routine complaint heard from anticoagulated patients seen at anticoagulation clinics, particularly female patients. It is described in connection with use of parenteral anticoagulants (unfractionated heparin [UFH] and low molecular weight heparin [LMWH]) and oral anticoagulants (vitamin K antagonists [VKA] and oral direct thrombin inhibitors and direct factor Xa inhibitors). The etiopathogenesis of this hair loss is still uncertain, but it may be a consequence of drug interactions or other underlying diseases that act in synergy, provoking alopecia. In view of this, a review of literature on the mechanisms involved in genesis of hair loss related to anticoagulation and current treatments was conducted at the anticoagulation clinic run by the Angiology Service at the Hospital Universitário Pedro Ernesto da Universidade do Estado do Rio de Janeiro (UERJ), in Rio de Janeiro, Brazil.

## METHODS

Searches were run for studies published from 1988 to 2019 in publications listed on the PubMed, MEDLINE, BIREME, and LILACS databases using the following keywords: alopecia, anticoagulation, adverse effects, and anticoagulants. The search returned a total of 17 articles selected and compiled, covering the pathophysiologic mechanisms of this complication and the therapeutic options.

According to reports registered on the Vigia Access^1^ database that describe cases of alopecia induced by anticoagulants, all anticoagulant agents can produce signs and symptoms of hair disease, of which alopecia is the most common, although the exact mechanisms remain unknown.^1^^,^^2^ All of the anticoagulants provoke a similar pattern of hair loss, with diffuse loss of hair with onset, in general, 2 to 4 months after starting anticoagulant use.^3^

### Hair physiology

The cycle of hair growth consists of three sequential and dynamic phases. In the anagen or long growth phase, continuous growth and vigorous mitotic activity occur in the hair matrix, lasting months or years and controlling hair length. The next phase is the catagen phase, or the brief apoptotic transition of the hair matrix cells, during which hair growth ceases and the follicle shortens, and the mature hair moves upwards towards the scalp. Finally, the telogen phase, is a short period when the hair is stationary, lasting approximately 3 months, during which the hair is at rest, awaiting removal by mechanical forces or substitution by new follicles in growth. Between 85 and 90% of hairs are in the anagen phase at any time, 9 to 14% are in the telogen phase, and 1% are in the catagen phase. There is significant variability between individuals in relation to the proportions of hair in each phase and their durations. At the end of the telogen phase, the hair falls and a new hair begins to grow from the follicle, restarting the cycle. Normally, around 50 to 100 hairs reach the telogen phase per day and fall. When more than 100 hairs per day reach the stationary phase, hair loss is observed (telogen effluvium). Interruption of the growth phase causes abnormal loss of anagen hairs (anagen effluvium).^1^^-^7

### Pathophysiology of alopecia

Alopecia is a condition characterized by hair loss, especially from the scalp. It can be classified as focal or diffuse and may involve abnormal scarring. When secondary to use of medications, particularly anticoagulants, alopecia is classified as diffuse and non-scarring. In these cases, it encompasses telogen effluvium with increased numbers of hairs entering the stationary phase.^2^^,^^3^^,^^5^

Drug-induced hair loss occurs via two different mechanisms, affecting the anagen and telogen phases. Anagen effluvium consists of abrupt cessation of hair growth caused by antimitotic activity and is often seen in connection with use of cytotoxic agents, such as chemotherapy drugs. Normally, this type of alopecia has rapid onset and anagen hairs start to fall out days or weeks after exposure. In contrast, telogen effluvium is the result of premature displacement of anagen hairs into the catagen phase, followed by the telogen phase. The visible effects usually appear some months after onset of the condition and may be secondary to certain situations, such as delivery, malnutrition, fever, surgery, and hemorrhage, and also to use of medications such as amlodipine, atorvastatin, angiotensin-converting enzyme inhibitors (ECA), and hydrochlorothiazide.^2^^,^^3^^,^^5^^,^^6^

## DISCUSSION

### Alopecia associated with use of anticoagulants

Use of anticoagulants is associated with a range of complications, of which bleeding has naturally been studied most. Other complications of lesser severity, such as alopecia, are little investigated or even ignored during anticoagulant therapy, even those that are common complaints among patients.^8^^-^10 Anticoagulants act on the anagen phase of hair growth, stimulating the follicle to prematurely enter the telogen phase, generally leading to visible alopecia around 2 to 4 months after administration is initiated.^2^^,^^5^^,^^7^^,^^8^

One hypothesis is that anticoagulant-induced alopecia might be caused by disseminated thrombosis in the microcirculation of capillaries that feed the roots of the hair, but this has not been proven scientifically.^4^ Another hypothesis, based on scalp biopsies, is that there may be distension of bundles of the dermis caused by bleeding with focal degeneration of collagen bundles, which would provoke strangulation of the hair root and injury to the connective tissue of the dermal papilla.^3^ Heparins and AVKs are the anticoagulants associated with this condition, with reported incidence rates from 30 to 40% with AVKs and 54 to 66% with UFH.^7^

Hair loss secondary to use of anticoagulants normally has onset in the frontotemporal area, later extending to the remainder of the scalp. However, it may continue even when anticoagulation is withdrawn, taking up to 3 months for spontaneous regrowth of hair to begin.^4^^,^^7^

### Warfarin

Warfarin is a VKA available in Brazil. It can induce hair follicles to enter the telogen phase prematurely, without interfering with the catagen phase. Based on the time and presentation of hair loss reported in connection with this drug, it is believed that the probable process of alopecia in these cases is associated with telogen effluvium.^5^^,^^6^^,^^8^^,^^9^ The occurrence of alopecia clinically associated with warfarin is common and is more frequent among female patients, with overall reported incidence from 30 to 40%.^1^^,^^2^^,^^7^^,^^9^^,^^10^ Characteristics such as older patient age, longer duration of treatment or higher dosage are risk factors associated with the condition and the majority of reports reveal that alopecia is reversed when treatment is withdrawn.^5^^,^^11^^-^14

### Heparins

The heparins (UFH and LMWH) are known to have antimitotic activity. However, other mechanisms may also be involved in the genesis of alopecia associated with this class of anticoagulant, since it has also been linked with changes such as thickening of the dermoepidermal junction, and has shown inhibitory effects on hair growth (suppression of proliferation of the epithelial bulb).^1^^-^3^,^^5^ Reported incidence rates of UFH-related alopecia range from 54 to 66%.^7^

### Direct oral anticoagulants (DOACs): direct factor Xa or IIa inhibitors

A prospective registry of 938 patients receiving anticoagulant treatment with rivaroxaban or dabigatran for atrial fibrillation or venous thromboembolism (the Dresden Registry)^12^ observed an incidence of spontaneously reported alopecia of 4.4 per 100 patients-year, at a mean time of 68 ± 76 days after administration of these medications and in all cases the patients were female.

### Treatment

Before attributing the cause of alopecia to anticoagulant use, it is important to investigate the dermatological condition in general. Certain comorbidities and use of other medications are frequently involved in the etiopathogenesis of telogen effluvium. Therefore, endocrine diseases, systemic diseases, prolonged fever, stress, weight loss, anemia, iron and vitamin D deficiencies, and inflammatory scalp disorders should all be investigated.^13^^-^16 It is important to take into consideration events and medications introduced 2 to 3 months before appearance of the condition. It should be emphasized that alopecia induced by anticoagulation can very often be superimposed over an underlying condition, such as androgenetic alopecia, aggravating hair loss, but not necessarily constituting its only cause.^13^^-^16 Along the same lines, withdrawal of oral contraception when indicated is another possible cause of telogen effluvium, regardless of whether anticoagulants are being used. It is therefore expected that patients taking an anticoagulant who substitute oral contraception with an intrauterine device will have more significant hair loss for a transitory period.^13^^-^15 Concomitant conditions that could aggravate the condition should be investigated objectively and treated.

Since 1988, a topical solution of minoxidil has been approved exclusively for treatment of androgenetic alopecia,^16^ because its use is not yet scientifically confirmed for alopecia with other etiologies, although the solution is nevertheless widely used in clinical practice. In 2019, a publication in the American Journal of Clinical Dermatology^16^ reviewed other potential indications for topical minoxidil to treat non-androgenetic alopecias, emphasizing the need for further studies to confirm efficacy for other types of alopecia. Minoxidil solution is available in several presentations, including 2 or 5% solution and 5% foam. It should be applied once or twice a day and response to treatment can occur up to 6 months after starting use. Its adverse effects include facial hypertrichosis and contact dermatitis. The foam presentation, without propylene glycol, appears to cause less local irritation.^16^ Supplementation with nutraceuticals and multivitamins containing biotin, zinc, vitamins A, C, E, and B complex and folic acid can be of use, primarily for patients who have specific deficiencies of these elements.^13^^-^16

In common with alopecia caused by other medications, the definitive treatment for alopecia induced by anticoagulants is definitive withdrawal of the medication. For patients who have indications for use of the anticoagulant for a limited time, this fact should be of comfort. However, many people need to take anticoagulants for extended periods, and alternative and palliative measures can and should be offered. Once other causes have been ruled out and the alopecia presented has been diagnosed as secondary to anticoagulation, the treating physician should discuss the benefits and need to continue the anticoagulant medication for a specific time with the patient. Patients who will be taking the anticoagulant for shorter periods, from 3 to 6 months, may not need a specific therapeutic intervention, but simply explanations and guidance. The patient will thus be more confident and relaxed about coping with this adverse effect, minimizing its repercussions for quality of life.

## CONCLUSIONS

Alopecia is a common complication of anticoagulant therapy that occurs with all types of anticoagulants at varying incidences, with onset from the first to sixth month of treatment. The condition causes hair loss in the frontotemporal region, which later extends to the remainder of the scalp, but which, in general, reverts after withdrawal of the medication. When there are indications for prolonged anticoagulation, other conditions that are also associated with alopecia should be investigated and adjuvant dermatological treatment should be considered.
