# Supplementary material for: Alopecia in anticoagulated patients
Source: J Vasc Bras. 2020 Jun 8;19:e20190018. doi: 10.1590/1677-5449.190018 (PMC8202206; doi:10.1590/1677-5449.190018)
Supplement: Translation

# Translation: Alopecia em pacientes anticoagulados

**Authors:** Marcos Arêas Marques, Carmen Lucia Lascasas Porto, Ana Letícia de Matos Milhomens, Juliana de Miranda Vieira, Claudia Carvalho Alcântara Gomes, Ana Thereza Cavalcanti Rocha, Cíntia da Silva Miller

**Affiliations:** 1 Universidade do Estado do Rio de Janeiro – UERJ, Hospital Universitário Pedro Ernesto, Unidade Docente Assistencial de Angiologia, Rio de Janeiro, RJ, Brasil.; 2 Hospital Barra D’Or, Dermatologia, Rio de Janeiro, RJ, Brasil.; 3 Faculdade de Medicina da Bahia, Departamento de Saúde da Família, Salvador, BA, Brasil.

**Keywords:** alopecia, efeitos colaterais e reações adversas associados a medicamentos, varfarina, heparina, anticoagulantes, rivaroxabana

## Abstract

A alopecia é uma complicação comum da terapia anticoagulante que pode ter repercussões psicológicas importantes nos pacientes, especialmente nos do sexo feminino, e interferir na decisão de extensão da anticoagulação. Esta revisão tem como objetivo descrever os mecanismos potencialmente envolvidos na gênese da alopecia durante a terapia anticoagulante, pois eles ainda não estão totalmente esclarecidos, e as terapias existentes, para a adoção das condutas mais adequadas.

## INTRODUÇÃO

A queixa de queda de cabelo em pacientes anticoagulados é rotineira nos ambulatórios de anticoagulação, principalmente em pacientes do sexo feminino, e é descrita com o uso de anticoagulantes parenterais [heparina não fracionada (HNF) e heparina de baixo peso molecular (HBPM)] e orais [antagonistas da vitamina K (AVK) e inibidores orais diretos da trombina e do fator X ativado]. A etiopatogenia da queda capilar ainda é incerta, mas pode ser consequente à**s** interações medicamentosas ou a outras doenças de base que atuam sinergicamente, provocando a alopecia. Mediante a este fato, no ambulatório de anticoagulação do Serviço de Angiologia do Hospital Universitário Pedro Ernesto da Universidade do Estado do Rio de Janeiro (UERJ), Rio de Janeiro (RJ), Brasil, foi realizada uma revisão da literatura sobre os mecanismos envolvidos na gênese da queda capilar relacionada à anticoagulação e os tratamentos atuais.

## MÉTODOS

Foi realizada uma busca em estudos publicados no período de 1988 a 2019, em publicações nas bases de dados PubMed, MEDLINE, BIREME e LILACS, com os seguintes descritores: alopecia, anticoagulação, efeitos adversos e anticoagulantes. A pesquisa totalizou 17 artigos selecionados e compilados, abordando os mecanismos fisiopatológicos dessa complicação e suas possibilidades terapêuticas.

De acordo com relatórios na base de dados Vigia Access^1^ sobre casos de alopecia induzida por anticoagulantes, todos os agentes podem provocar sinais e sintomas de doença capilar, sendo a alopecia o mais comum, embora seus mecanismos exatos permaneçam desconhecidos^1^^,^^2^. Todos os anticoagulantes provocam um padrão de queda capilar semelhante, com perda difusa dos cabelos iniciando-se, em geral, 2 a 4 meses após o início do uso^3^.

### Fisiologia capilar

O ciclo de crescimento capilar compreende três fases sequenciais e dinâmicas. Na fase anágena, ou de crescimento longo, ocorre crescimento contínuo e atividade mitótica vigorosa na matriz capilar que perdura por meses ou anos, determinando seu comprimento. A seguir, ocorre a fase catágena, ou apoptótica transicional breve da matriz da célula capilar, na qual o crescimento capilar cessa, havendo um encurtamento do folículo, e o cabelo maduro se move para cima em direção ao couro cabeludo. Por último, ocorre a fase telógena, curta e estacionária dos fios, que dura aproximadamente 3 meses e, durante este período, o cabelo repousa, aguardando sua retirada por forças mecânicas ou substituição por novos folículos em crescimento. Entre 85 e 90% dos cabelos em qualquer momento estão na fase anágena, 9 a 14% estão na fase telógena e 1% está na fase catágena. Existe significativa variabilidade entre indivíduos em relação à proporção de cabelo e duração de cada fase. Ao término da fase telógena, o cabelo cai e um novo começa a crescer no folículo, reiniciando o ciclo. Normalmente, cerca de 50 a 100 fios de cabelo alcançam a fase telógena por dia e caem. Quando mais de 100 fios por dia alcançam a fase estacionária, observa-se queda de cabelos (eflúvio telógeno). A interrupção da fase de crescimento causa perda anormal dos cabelos anágenos (eflúvio anágeno)^1^^-^7.

### Fisiopatologia da alopecia

A alopecia é uma condição em que há perda de pelos, especialmente do couro cabeludo. Ela pode ser classificada como focal ou difusa e pode cursar com a presença de cicatrizes anormais. Quando secundária ao uso de medicamentos, particularmente anticoagulantes, a alopecia é classificada como difusa e não cicatricial. Neste contexto, engloba o eflúvio telógeno com o aumento do número de fios de cabelo que entram na fase estacionária^2^^,^^3^^,^^5^.

A perda de cabelo induzida por drogas ocorre por dois mecanismos diferentes, afetando as fases anágena e telógena. O eflúvio anágeno representa a cessação abrupta do crescimento capilar por atividade antimitótica e é visto com frequência com o uso de agentes citotóxicos, como os quimioterápicos. Normalmente, o início deste tipo de alopecia é rápido, e os pelos anágenos caem dentro de dias a semanas após a exposição. Já o eflúvio telógeno resulta do deslocamento prematuro de cabelos anágenos para a fase catágena, seguindo para a fase telógena. Os efeitos visíveis geralmente aparecem alguns meses após o início do quadro e podem ser secundários a algumas situações, como parto, desnutrição, febre, cirurgia e hemorragia, bem como ao uso de medicamentos como amlodipina, atorvastatina, inibidores da enzima conversora de angiotensina (ECA) e hidroclorotiazida^2^^,^^3^^,^^5^^,^^6^.

## DISCUSSÃO

### Alopecia associada ao uso de anticoagulantes

O uso de anticoagulantes está associado a diversas complicações, sendo o sangramento naturalmente a mais estudada. Outras complicações de menor gravidade, como a alopecia, mesmo sendo uma queixa frequente dos pacientes, são pouco abordadas ou mesmo negligenciadas durante o período de terapia anticoagulante^8^^-^10. Os anticoagulantes agem na fase anágena do crescimento capilar, estimulando o folículo capilar a entrar na fase telógena prematuramente, levando à alopecia visível comumente 2 a 4 meses após o início de sua administração^2^^,^^5^^,^^7^^,^^8^.

Uma hipótese é que a alopecia induzida por anticoagulantes poderia ocorrer devido à trombose disseminada na microcirculação dos capilares que irrigam as raízes dos fios de cabelo, porém ela ainda não tem comprovação científica^4^. Outra hipótese aventada a partir de exames de biópsia de couro cabeludo é que poderia ocorrer distensão dos feixes da derme por sangramento com degeneração focal peculiar dos feixes colágenos, e isto provocaria um estrangulamento da raiz do cabelo e lesão do tecido conectivo da papila dérmica^3^. As heparinas e os AVKs são os anticoagulantes associados a esta condição, com incidências relatadas entre 30 e 40% com AVKs e 54 a 66% com HNF^7^.

A perda capilar secundária ao uso de anticoagulantes normalmente se inicia na região frontotemporal, estendendo-se posteriormente pelo resto do couro cabeludo. No entanto, pode continuar mesmo com a suspensão da anticoagulação, levando até 3 meses para o início da recapilarização espontânea^4^^,^^7^.

### Varfarina

A varfarina, um AVK disponível no Brasil, induz os folículos capilares a entrarem na fase telógena precocemente, não interferindo na fase catágena. Baseado no tempo e na apresentação da perda de cabelos reportada em relação a esta droga, acredita-se que o processo provável de alopecia nestes casos está associado ao eflúvio telógeno^5^^,^^6^^,^^8^^,^^9^. A incidência de alopecia clinicamente associada à varfarina é comum, mais frequente em pacientes do sexo feminino e com incidências gerais relatadas entre 30 e 40%^1^^,^^2^^,^^7^^,^^9^^,^^10^. Características como idade mais avançada do paciente, duração mais prolongada do tratamento ou dosagem maior são fatores de risco associados e a maioria dos relatos revela que a alopecia é revertida com a interrupção do tratamento^5^^,^^11^^-^14.

### Heparinas

As heparinas (HNF e HBPM) sabidamente apresentam atividade antimitótica. Outros mecanismos, no entanto, também podem estar envolvidos na gênese da alopecia associada a esta classe de anticoagulante, já que esta tem demonstrado alterações como o aumento da junção dermoepidérmica, bem como demonstra ter efeitos inibitórios sobre o crescimento do cabelo (supressão da proliferação do bulbo epitelial)^1^^-^3^,^^5^. As incidências relatadas de alopecia com HNF são de 54 a 66%^7^.

### Anticoagulantes orais diretos (DOACs): inibidores diretos do fator Xa ou IIa

Um registro prospectivo de 938 pacientes em tratamento anticoagulante para fibrilação atrial ou tromboembolismo venoso com rivaroxabana ou dabigatrana (*Dresden Registry*)^12^ observou uma incidência de alopecia espontaneamente relatada de 4,4 por 100 pacientes-ano, em um tempo médio de 68 ± 76 dias após a administração destes medicamentos, sendo todos os casos em pacientes do sexo feminino.

### Tratamento

Antes de atribuir a causa da alopecia ao uso de anticoagulante, é fundamental a investigação do quadro dermatológico de modo global. Algumas comorbidades e o uso de outras medicações estão frequentemente implicados na etiopatogenia do eflúvio telógeno. Desta forma, devem ser pesquisados endocrinopatias, doenças sistêmicas, febre prolongada, estresse, perda de peso, anemia, deficiência de ferro e vitamina D e desordens inflamatórias do couro cabeludo^13^^-^16. Nesta investigação, é importante considerar eventos e medicamentos introduzidos 2 a 3 meses antes do início do quadro. Vale ressaltar que, muitas vezes, a alopecia induzida pela anticoagulação pode se superpor a um quadro de base, como alopecia androgenética, levando ao agravamento da queda de cabelos, não, obrigatoriamente, representando a sua única causa^13^^-^16. Da mesma forma, a interrupção do anticoncepcional oral, quando indicada, por si só já representa uma possível causa para a ocorrência do eflúvio telógeno, independente do uso do anticoagulante. Assim, é esperado, nas pacientes em uso de anticoagulante e que substituam o anticoncepcional oral pelo dispositivo intrauterino (DIU), que a queda capilar seja mais significativa por um período transitório^13^^-^15. Condições concomitantes que possam agravar o quadro devem ser objetivamente investigadas e tratadas.

Desde 1988, a solução tópica de minoxidil foi aprovada exclusivamente para o tratamento da alopecia androgenética^16^, pois sua aplicação ainda não está cientificamente comprovada em quadros de alopecia de outras etiologias, embora esta solução seja amplamente utilizada na prática clínica. Em 2019, uma publicação no *American Journal of Clinical Dermatology* 16 revisou outras indicações potenciais do minoxidil tópico nas alopecias não androgenéticas, reforçando a necessidade da realização de novos estudos para comprovar a eficácia em outros tipos de alopecia. A solução de minoxidil está disponível em várias formulações, incluindo solução a 2 ou 5% e espuma a 5%, devendo ser aplicada uma ou duas vezes ao dia, e a resposta ao tratamento pode ocorrer em até 6 meses após sua introdução. Entre seus efeitos adversos, destacam-se hipertricose facial e dermatite de contato. A formulação em espuma, sem propilenoglicol, parece causar menos irritação local^16^. A suplementação com nutracêuticos e polivitamínicos contendo biotina, zinco, vitamina A, C e E, complexo B e ácido fólico pode ser útil, sobretudo em pacientes que apresentem deficiências específicas desses elementos^13^^-^16.

Da mesma forma que para a alopecia causada por outras medicações, o tratamento definitivo para a alopecia induzida por anticoagulantes seria a retirada definitiva deste medicamento. Para aqueles pacientes que têm indicação do uso do anticoagulante por tempo limitado, este deve ser um fato tranquilizador. No entanto, muitos necessitam do uso estendido de anticoagulantes, e medidas alternativas e paliativas podem e devem ser oferecidas. Uma vez excluídas outras causas e diagnosticado que a alopecia apresentada é, de fato, secundária à anticoagulação, os benefícios e a necessidade da manutenção da medicação anticoagulante por um tempo determinado devem ser claramente discutidos entre o paciente e o médico assistente. Pacientes que farão uso do anticoagulante por períodos mais curtos, entre 3 e 6 meses, podem não necessitar de uma intervenção terapêutica específica e, sim, apenas de esclarecimento e orientação. Dessa forma, o paciente terá mais segurança e tranquilidade para lidar com esse efeito adverso e minimizar a repercussão disso na sua qualidade de vida.

## CONCLUSÃO

A alopecia é uma complicação comum da terapia anticoagulante, ocorrendo com todos os tipos de anticoagulantes em incidências diferentes, iniciando-se do primeiro ao sexto mês de tratamento. Esta condição causa uma perda na região frontotemporal, estendendo-se posteriormente pelo resto do couro cabeludo, mas que, em geral, reverte após a cessação do uso da medicação. Quando há indicação de anticoagulação prolongada, devem ser afastadas outras condições também associadas a alopecia e ponderado tratamento dermatológico adjuvante.
